# Analysis of Laparoscopic and Transvaginal Ovarian Drilling on Polycystic Ovarian Syndrome: A Narrative Review of Outcomes

**DOI:** 10.7759/cureus.103930

**Published:** 2026-02-19

**Authors:** Mah Rukh Afzal, Mohamed Alassar, Ariba Furqan, Linh Huynh, Maher Feras Alnajjar, Safa Gismalla, Ibrahim Mostafa Hussein, Adel Mahmah

**Affiliations:** 1 Surgery, Allama Iqbal Medical College, Lahore, PAK; 2 General Practice, Bahçeşehir Üniversitesi Medical Park, Istanbul, TUR; 3 General Practice, King Salman Armed Forces Hospital, Tabuk, SAU; 4 Medicine, Civil Hospital Karachi, Karachi, PAK; 5 Osteopathic Medicine, University of California, Irvine, USA; 6 Medicine, Zagazig University, Zagazig, EGY; 7 Medicine and Health Sciences, Omdurman Islamic University, Omdurman, SDN; 8 Medicine, Newgiza University, Cairo, EGY; 9 General Practice, Bahçeşehir University, Istanbul, TUR

**Keywords:** gynecology surgery, laproscopic drilling, narrative review, polycystic ovary syndrome (pcos), transvaginal ovarian drilling

## Abstract

The primary objective of polycystic ovarian syndrome (PCOS) treatment is to achieve mono-ovulatory cycles, thereby enabling pregnancy and childbirth. Laparoscopic ovarian drilling (LOD) and transvaginal ovarian drilling (TVOD) are widely used second-line treatments for ovulation induction in PCOS patients who do not respond to clomiphene citrate (CC). LOD involves the application of heat or laser energy to create small perforations in the ovary, whereas TVOD involves ultrasound-guided ovarian puncture. Both LOD and TVOD have been shown to be effective in promoting ovulation and enhancing fertility outcomes in PCOS patients. TVOD offers several potential advantages over LOD, including a lower risk of surgical complications, reduced cost, and the ability to be performed in an outpatient setting. Both LOD and TVOD are effective treatment options for PCOS-related infertility. The choice between the two procedures should be based on individual patient characteristics, including body mass index (BMI), hormonal profiles, and previous responses to treatment. Further research is needed to optimize these techniques and clarify their clinical applications. Patients with clomiphene-resistant PCOS should be carefully assessed for both LOD and TVOD. LOD may be more suitable for patients with elevated luteinizing hormone (LH) levels and shorter durations of infertility. In contrast, TVOD may be preferred for patients who would benefit from a less invasive approach with a lower risk of adhesion formation.

## Introduction and background

Polycystic ovarian syndrome (PCOS) is one of the most common endocrine and metabolic disorders and a leading cause of anovulatory infertility, with a reported prevalence of 6%-20% among women of reproductive age, accounting for more than 80% of infertility cases due to anovulation. It is characterized by a heterogeneous combination of clinical features (anovulation and hyperandrogenism), biochemical abnormalities (elevated serum luteinizing hormone (LH) and androgen levels), and ovarian morphological features (polycystic ovaries). The induction of mono-ovulatory cycles is the primary goal of treatment [1-3].

Clomiphene citrate (CC) is the first-line therapy for ovulation induction in women with PCOS. When CC treatment fails, defined as the failure to achieve ovulation after six months of therapy at an appropriate dose or failure to conceive, patients are considered clomiphene resistant [4]. Several studies have demonstrated a systemic low-grade chronic inflammatory state in women with PCOS, characterized by increased levels of cytokines, including interleukin (IL)-6, IL-18, and tumor necrosis factor-α (TNF-α), as well as acute-phase proteins such as high-sensitivity C-reactive protein (hs-CRP) and heat shock protein 70 (HSP70) in the peripheral circulation [5]. Although patients with PCOS typically produce an increased number of oocytes, these oocytes are often of poor quality, resulting in lower fertilization rates [6].

Laparoscopic ovarian drilling (LOD) and transvaginal ovarian drilling (TVOD) are considered effective second-line treatment options for approximately 15%-40% of women with clomiphene-resistant PCOS. LOD involves the use of thermal energy or laser during laparoscopy to create small perforations in the ovarian cortex, thereby facilitating follicular growth and ovulation, and has demonstrated clinical efficacy in improving pregnancy and live birth rates. In contrast, TVOD is a novel minimally invasive surgical approach that uses a sharp needle under transvaginal ultrasound guidance to puncture the ovaries, facilitating ovum release and aspiration of small follicles. This procedure results in a reduction in intraovarian and serum androgen levels, inhibin B, LH, and other steroid hormones, thereby restoring the hypothalamic-pituitary-ovarian feedback mechanism and promoting appropriate gonadotropin secretion and follicular development. Additional mechanisms contributing to ovulation restoration include improved ovarian blood flow, enhanced delivery of gonadotropins and local growth factors, and improved insulin sensitivity following ovarian drilling. Compared with LOD, TVOD offers several potential advantages, including a lower risk of surgical complications, particularly iatrogenic adhesions and premature ovarian failure, avoidance of ovarian hyperstimulation syndrome and multiple pregnancies, reduced procedural costs, and the ability to be performed in an outpatient setting [1-3]. Furthermore, a single LOD procedure may result in repeated physiological ovulatory cycles and subsequent pregnancies without the need for repeated medical interventions. Anti-Müllerian hormone (AMH) is widely accepted as a reliable marker of ovarian reserve, and several studies have reported a significant reduction in serum AMH levels following LOD. Given its minimal inter- and intracycle variability, serum AMH concentration is considered particularly useful for detecting subtle changes in ovarian reserve after ovarian drilling [3]. This article aims to provide an overview of the effects of LOD on fertility outcomes in women with PCOS, with comparative consideration of transvaginal approaches.

## Review

This section outlines the methodological approach used to conduct a narrative review comparing the outcomes of LOD and TVOD in the treatment of PCOS. The review evaluated the effectiveness, safety, and reproductive outcomes associated with both procedures.

Literature search strategy

A comprehensive literature search was conducted using PubMed, Scopus, and the Cochrane Library. The search strategy included keywords such as “laparoscopic ovarian drilling,” “transvaginal ovarian drilling,” “PCOS treatment,” “infertility,” “ovulation induction,” and “clinical outcomes.” Studies published in English between January 2000 and March 2023 were considered eligible for inclusion.

Inclusion and exclusion criteria

Inclusion criteria included comparative studies evaluating LOD and TVOD. Eligible studies reported outcomes related to ovulation rates, pregnancy rates, complication rates, and changes in hormonal profiles. Study designs encompassed randomized controlled trials, cohort studies, case-control studies, and observational studies.

Exclusion criteria included studies that did not directly compare LOD and TVOD. Case reports and review articles lacking primary data were excluded, as were studies without clearly defined outcome measures.

Data extraction and quality assessment

Data from eligible studies were extracted using a standardized data collection form, focusing on study design, sample size, patient demographics, procedural details, and reported outcomes. Two independent reviewers assessed the quality of included studies using criteria adapted from the Newcastle-Ottawa Scale for nonrandomized studies and the Cochrane Risk of Bias Tool for randomized controlled trials [7].

Data analysis

The extracted data were synthesized narratively, with emphasis on comparing the efficacy and safety profiles of LOD and TVOD. Outcomes of interest included ovulation rates, pregnancy rates, time to pregnancy, hormonal changes, and procedure-related complications. Patient-reported outcomes and quality-of-life indicators were also considered when available.

Ethical considerations

As this study represents a narrative review of previously published literature, ethical approval was not required. The review was conducted in accordance with ethical standards for research reporting.

Laparoscopic ovarian drilling (LOD)

When first-line ovulation induction therapies fail to induce ovulation in women with PCOS, LOD may be considered as an alternative second-line treatment [8,9]. A stepwise approach to PCOS-related infertility enables the majority of patients to achieve pregnancy and deliver a healthy child, with the primary objective being the induction of mono-ovulatory cycles [2]. Laparoscopy-associated techniques are regarded as the most conventional surgical approach and can be performed under general anesthesia in a standard operating room. The procedure allows the use of laser energy or multiple ovarian perforations via monopolar electrocoagulation, as well as limited ovarian biopsies. In addition, laparoscopy may reveal significant pelvic pathology, potentially resulting in a modification of the therapeutic strategy. LOD may be considered for women with CC-resistant PCOS, particularly when there are contraindications to multiple pregnancies, an increased risk of multifetal gestation, or additional indications for laparoscopy [2,4]. As this intervention typically requires only a single surgical session, LOD has been shown to reduce both direct and indirect treatment costs in CC-resistant PCOS patients [10]. Commonly, three to eight diathermy punctures delivering 600-800 J of energy per ovary are performed, resulting in the restoration of regular ovulation in approximately 74% of patients within three to six months. However, performing more than eight punctures has been associated with an increased risk of postoperative pelvic adhesions and a reduction in ovarian reserve. In contrast, a well-designed randomized study demonstrated that LOD was more costly and less effective than metformin in certain patient populations [11].

Fernandez et al. reported in a comprehensive review that ovarian drilling resulted in spontaneous restoration of fertility in 20%-64% of women with PCOS who were previously infertile due to anovulation and resistant to CC therapy [4]. Similarly, Campo’s meta-analysis demonstrated success rates ranging from 44% to 50% [12]. LOD appears to be most effective in patients with elevated luteinizing hormone (LH) levels (>10 IU/L) and a duration of infertility of less than three years. Evidence from 38 trials indicates that the live birth rate following medical ovulation induction alone is approximately 42%, whereas rates following LOD ranged from 28% to 40%. Importantly, LOD is associated with a significant reduction in multiple pregnancy rates (odds ratio, 0.34; 95% confidence interval, 0.18-0.66), decreasing the risk from 5.0% to between 0.9% and 3.4% [13]. These acceptable pregnancy outcomes, combined with advancements in laparoscopic techniques, have contributed to LOD becoming a widely performed procedure worldwide [9].

In addition to reproductive benefits, LOD has been associated with improvements in metabolic parameters, including a reduction in the prevalence of dyslipidemia, type 2 diabetes mellitus, and metabolic syndrome, thereby potentially lowering long-term cardiovascular risk in women with PCOS [14,15]. Prognostic outcomes are most favorable in lean patients with elevated serum LH levels [16], and studies have demonstrated that women who ovulate after LOD exhibit higher pretreatment LH concentrations compared with nonresponders [17]. Furthermore, serum androgen levels have been shown to gradually decline over time following LOD, possibly reflecting age-related hormonal changes [18].

Although the primary goal of LOD is to reduce excessive androgen levels, overcorrection may occur, potentially resulting in abnormally low androgen concentrations. Clinical manifestations of this hormonal imbalance may include fatigue, muscle weakness, and mood disturbances. Nevertheless, a significant postoperative reduction in serum testosterone levels following LOD has been consistently observed and is associated with improvements in ovulatory function and fertility outcomes [19]. Table 1 summarizes the effects of LOD on hormonal parameters and clinical symptoms in women with PCOS (n = 50) [20].

**Table 1:** Effect of LOD on hormonal parameters and clinical outcomes LOD: laparoscopic ovarian drilling; LH: luteinizing hormone; FSH: follicle-stimulating hormone; AMH: anti-Müllerian hormone

Parameter	Before LOD	After LOD
LH	Elevated	Significantly reduced
FSH	Normal/low	Improved
LH/FSH ratio	Increased	Reduced
Serum testosterone	Elevated	Decreased
AMH	High	Reduced
Ovulation rate	Low/absent	Increased
Menstrual regularity	Irregular	Improved
Fertility outcome	Anovulatory infertility	Increased pregnancy rate

Safety and Complications of LOD

One of the main limitations of LOD is the risk of iatrogenic adhesion formation, which may result from excessive hemorrhage from the ovarian surface or early postoperative contact between the ovary and surrounding tissues following cauterization. Another potential complication is premature ovarian failure (POF), particularly in cases where the ovarian blood supply is inadvertently compromised or when excessive numbers of punctures are performed, leading to significant depletion of the ovarian follicular pool or the formation of anti-ovarian antibodies, which may result in early menopause and infertility. To date, only one documented case of ovarian atrophy has been reported following high-energy drilling, involving eight coagulation sites at 400 W for five seconds. When performed using appropriate technique and controlled energy settings, LOD does not appear to pose a significant risk to ovarian reserve [21,22]. However, a rare case of unilateral ovarian atrophy following LOD has also been reported [10].

Transvaginal ovarian drilling (TVOD)

Efficacy

With continued advancements in transvaginal hydrolaparoscopy (THL), ovarian drilling can now be performed via an ultrasound-guided transvaginal approach as a day-surgery procedure [23]. Fernandez et al. were the first to describe THL under general anesthesia using bipolar electrosurgery as a novel approach to ovarian drilling in women with PCOS [24]. TVOD is primarily indicated for clomiphene-resistant PCOS and may be performed using THL with a watery distension medium [23,25].

TVOD involves ultrasound-guided puncture of the ovary to facilitate ovulation from the dominant follicle and aspiration of follicular fluid. This process results in a reduction in androgen and steroid hormone levels, leading to decreased LH concentrations and relative increases in follicle-stimulating hormone (FSH), thereby promoting follicular development and ovulation. Prior to TVOD, a bimanual vaginal examination and transvaginal ultrasound are required to assess uterine position and exclude pelvic pathology [26]. Reduction of intraovarian and serum LH levels through follicular fluid aspiration via ultrasound-guided transvaginal needle drilling (UTND) contributes to the rapid restoration of hypothalamic-pituitary feedback, with additional effects mediated by the removal of inhibin and other intraovarian factors [27].

Outcomes

Multiple studies have reported that transvaginal hydrolaparoscopic ovarian drilling (THLOD) achieves ovulation and conception rates comparable to laparoscopic approaches in women with PCOS [23,28,29]. In one study, endoscopic transvaginal ovarian capsule drilling was performed in 39 patients with a mean infertility duration of 26.5 ± 2.6 months. Patients were referred due to clomiphene resistance, failed gonadotropin-induced ovulation, or IVF failure, and 29 patients achieved pregnancy, with a mean interval of 7.2 ± 5.4 months between the procedure and conception. Postoperative evaluation conducted four to six weeks after the intervention demonstrated a reduction in LH levels from 15 ± 10 IU/L to 8 ± 3 IU/L, while FSH levels increased from 5.5 ± 2 IU/L to 6.1 ± 1.5 IU/L. Plasma testosterone levels also decreased from 1.7 ± 1.2 ng/mL to 1.1 ± 0.7 ng/mL, with significant reductions in LH and testosterone concentrations (p < 0.01) following the procedure [23].

A meta-analysis including five randomized controlled trials involving 639 women concluded that, among women with clomiphene-resistant PCOS, ovulation rates following TVOD ranged between 50.6% and 68.8% [26]. In addition, a case-control study of 123 women undergoing TVOD demonstrated significant postoperative reductions in serum AMH levels, ovarian volume, and power Doppler flow indices compared with preoperative values [30]. Furthermore, in a study of 11 women with PCOS undergoing assisted reproductive technology (ART), TVOD was associated with the need for higher FSH doses postprocedure compared with baseline (52.2 ± 15 IU vs. 33.5 ± 12 IU), resulting in a greater number of retrieved oocytes with similar estradiol levels. This was accompanied by significantly improved cleavage and fertilization rates (66% vs. 27% and 72% vs. 54%, respectively), while pregnancy and implantation rates were comparable to those of normo-ovulatory women undergoing IVF for tubal factor infertility [31]. Predictors of successful outcomes following LOD are summarized in Table 2 [21].

**Table 2:** Predictors of success of LOD LOD: laparoscopic ovarian drilling; BMI: body mass index; LH: luteinizing hormone

Predictor	Association with outcome
LH > 10 IU/L	Higher ovulation and pregnancy rates
Duration of infertility < 3 years	Better response to LOD
Lean or normal BMI	Improved reproductive outcome
Younger age	Higher success rate
Lower AMH levels	Better ovarian response
Absence of severe hyperandrogenism	Improved ovulation

Complications and Advantages of TVOD

Rectal perforation is a recognized but uncommon complication of transvaginal access. In addition, adhesions and endometriotic lesions located beyond the immediate vicinity of the vaginal apex may be difficult to access using this approach. To reduce the risk of bowel injury, prophylactic bowel preparation, including the use of a laxative and/or enema, may be considered prior to the procedure. In a large retrospective cohort study involving 2,288 surgical procedures, of which 374 included ovarian drilling, 23 patients (1%) experienced inability to access the Douglas pouch. The overall complication rate was 0.74%, primarily due to bleeding (n = 4) and intestinal perforation (n = 13) requiring laparoscopy. All cases of intestinal perforation were managed conservatively with antibiotics for six days, and no further complications were reported [32].

Despite these risks, the transvaginal approach for ovarian capsule drilling offers several advantages, including a favorable safety profile, particular benefit in obese patients, a reduced risk of adhesion formation, and a very low rate of procedure-related complications. Compared with LOD, THLOD is associated with a lower incidence of adhesions, a finding attributed to shorter operative times and the use of saline distension rather than pneumoperitoneum, which may reduce peritoneal irritation [33,34]. THLOD has also been proposed as a useful, minimally invasive, and cost-effective technique for improving ovarian response in PCOS patients with poor response to in vitro fertilization (IVF) [35]. Furthermore, this technique may be safely repeated in cases of recurrent anovulation or failed ovulation induction when well tolerated under conscious sedation. TVOD also allows for aspiration of small follicles, further contributing to the reduced risk of adhesion formation [31]. Overall, the transvaginal approach represents a less invasive and potentially more efficient alternative to standard LOD [23,25].

Discussion

Comparative Effectiveness of LOD and TVOD

LOD is an established treatment option for women with PCOS who are resistant to CC [36-38]. Following LOD, significant reductions have been observed in serum testosterone, free testosterone, LH, the LH/FSH ratio, insulin levels, and fasting glucose scores [39,40]. However, some studies have reported no significant changes in glucose levels or the glucose-to-insulin ratio [41].

**Figure 1:**
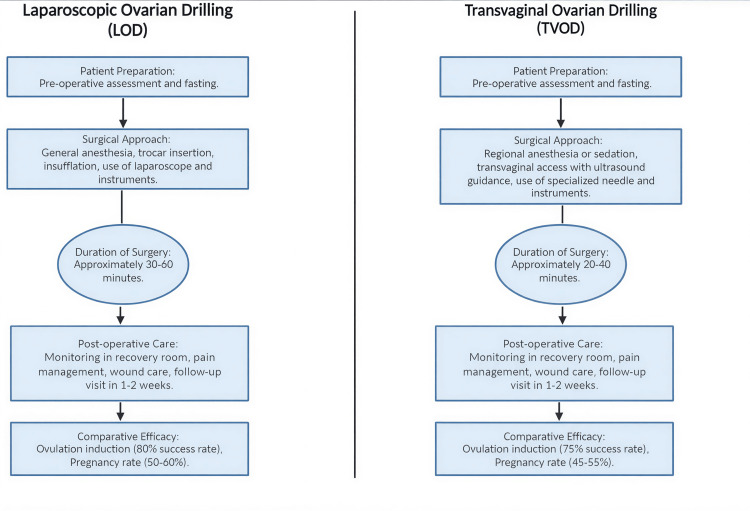
Comparative analysis of LOD and TVOD LOD: laparoscopic ovarian drilling; TVOD: transvaginal ovarian drilling

THL represents an alternative surgical approach, with studies demonstrating a lower risk of postoperative adhesion formation. However, evidence regarding its effect on menstrual cycle regularity remains limited. Both LOD and TVOD have been shown to reduce preoperative LH levels, AMH concentrations, and the LH/FSH ratio [42]. A study evaluating THL as a treatment for CC-resistant PCOS reported progressively increasing ovulation rates, reaching 64.1% at one month, 79.5% at three months, and 82.9% at six months postprocedure. During follow-up, 28 patients conceived, corresponding to a pregnancy rate of 70.1% [43]. A comparative analysis of LOD and TVOD is presented in Figure 1.

Strengths and Limitations of Each Technique

The principal advantages of LOD include shorter time to pregnancy, reduced reliance on ovulation induction medications, cost-effectiveness, and the potential for ambulatory performance [2]. Nevertheless, intraoperative challenges may arise due to general anesthesia, laparoscopy-related risks, and potential electrical injury. Postoperative complications include the risk of peri-adnexal adhesion formation and possible impairment of ovarian reserve [4]. Additionally, women with CC-resistant PCOS who are obese (body mass index (BMI): >25 kg/m²), have infertility lasting longer than three years, exhibit low basal LH levels (<10 IU/L), demonstrate marked biochemical hyperandrogenism, or have elevated basal AMH levels (≥7.7 ng/mL) may experience poorer reproductive outcomes following LOD [44].

Effects on Serum AMH and Antral Follicle Count

A commonly used biomarker to assess the effectiveness of PCOS interventions is the reduction in serum AMH levels [45]. Elevated AMH levels in PCOS have been associated with an increased antral follicle count or enhanced AMH production per follicle [46]. As a nonpharmacological intervention, LOD has been shown to significantly reduce AMH concentrations in women with PCOS [17,47], likely contributing to improved clinical outcomes through reductions in testosterone levels and normalization of the LH/FSH ratio [20,48].

Hyperprolactinemia Following Ovarian Cauterization

Hyperprolactinemia is a potential postoperative complication following LOD. Women who fail to ovulate after ovarian drilling despite improved hormonal profiles and reduced serum testosterone levels may exhibit elevated prolactin (PRL) concentrations. The underlying mechanism remains unclear, although one proposed explanation involves ovarian surface scarring and persistent neural stimulation, leading to neurogenic hyperprolactinemia [49].

Patient-Specific Factors in Surgical Decision-Making

Given the distinct advantages and limitations of each surgical technique, careful consideration of patient-specific factors is essential when selecting the most appropriate intervention. LOD may be preferred in women with CC-resistant PCOS who have contraindications to multiple pregnancies or additional indications for laparoscopy [2]. One notable advantage of ovarian drilling is its low risk of multiple gestations and ovarian hyperstimulation syndrome [4]. Other important determinants of surgical success include hormonal profile and BMI. Women with PCOS who have preoperative LH levels of >10 IU/L, a BMI of <30 kg/m², and ovulatory resistance to CC are considered optimal candidates for LOD [50]. A comparative overview of LOD and TVOD is illustrated in Figure 1. Table 3 shows the comparison of efficacy between LOD and other medical treatments in CC-resistant PCOS patients.

**Table 3:** Comparison between LOD and TVOD LOD: laparoscopic ovarian drilling; TVOD: transvaginal ovarian drilling

Feature	LOD	TVOD
Surgical approach	Laparoscopic	Transvaginal, ultrasound-guided
Anesthesia	General anesthesia	Local/conscious sedation
Invasiveness	Moderately invasive	Minimally invasive
Setting	Operating room	Outpatient/day surgery
Risk of adhesions	Higher	Very low
Cost	Higher	Lower
Recovery time	Longer	Shorter
Suitability for obese patients	Limited	More suitable
Ovulation rate	28-74%	50-69%
Pregnancy rate	28-40%	Up to 74%
Repeatability	Limited	Can be repeated

Figure 1 and Tables 1-3 were created entirely by the authors. No figures or tables were reproduced, adapted, or obtained from previously published sources.

## Conclusions

LOD represents a well-established second-line treatment for CC-resistant PCOS, with the primary goal of restoring mono-ovulatory cycles. When appropriately performed, LOD is a safe and effective procedure that can improve ovulation, pregnancy, and live birth rates while significantly reducing the risk of multiple gestations. Its benefits are most pronounced in carefully selected patients, particularly those with elevated LH levels and shorter durations of infertility. Although potential risks such as adhesion formation and ovarian reserve reduction exist, these complications are largely preventable with proper technique and energy control.

TVOD has emerged as a promising minimally invasive alternative to conventional laparoscopy, offering comparable efficacy with lower procedure-related morbidity, reduced adhesion risk, and suitability for outpatient or day-surgery settings. By improving hormonal profiles and facilitating ovulation, TVOD expands therapeutic options for women with PCOS-related infertility, particularly those who may benefit from a less invasive approach. The choice between LOD and TVOD should be individualized, taking into account patient characteristics, prior treatment response, and procedural risk profiles. Further research is warranted to optimize patient selection and refine both techniques to maximize reproductive outcomes.
